# Blood Mercury Reporting in NHANES: Identifying Asian, Pacific Islander, Native American, and Multiracial Groups

**DOI:** 10.1289/ehp.8464

**Published:** 2005-09-21

**Authors:** Jane M. Hightower, Ann O’Hare, German T. Hernandez

**Affiliations:** 1 Department of Internal Medicine, California Pacific Medical Center, San Francisco, California, USA; 2 Department of Medicine, Veterans’s Administration and University of California San Francisco, San Francisco, California, USA; 3 Department of Medicine, University of California San Francisco and San Francisco General Hospital, San Francisco, California, USA

**Keywords:** Alaskan Natives, American Medical Association, Asians, Centers for Disease Control, fish, mercury, methylmercury, multiracial, National Health and Nutrition Examination Survey, Native Americans, Pacific Islanders, reference dose, women

## Abstract

**Introduction:**

Asians, Pacific Islanders, and Native Americans are a potentially high-risk group for dietary exposure to methylmercury through fish consumption. However, blood mercury levels in this group have not been identified in recent reports of the National Health and Nutrition Examination Survey (NHANES) for the years 1999–2002.

**Methods:**

We used NHANES data from 1999–2002 to obtain population estimates of blood mercury levels among women of childbearing age classified as belonging to the “other” racial/ethnic group (Asian, Pacific Islander, Native American, and multiracial; *n* = 140). Blood mercury levels in this group were compared with those among all other women participants, classified as Mexican American, non-Hispanic black, non-Hispanic white, and “other” Hispanic.

**Results:**

An estimated 16.59 ± 4.0% (mean ± SE) of adult female participants who self-identified as Asian, Pacific Islander, Native American, or multiracial (*n* = 140) had blood mercury levels ≥5.8 μg/L, and 27.26 ± 4.22% had levels ≥3.5 μg/L. Among remaining survey participants (*n* = 3,497), 5.08 ± 0.90% had blood mercury levels ≥5.8 μg/L, and 10.86 ± 1.45% had levels ≥3.5 μg/L.

**Conclusions:**

Study subjects in NHANES who self-identified as Asian, Pacific Islander, Native American, or multiracial had a higher prevalence of elevated blood mercury than all other racial/ethnic participants in the survey. Future studies should address reasons for the high mercury levels in this group and explore possible interventions for lowering risk of methylmercury exposure in this population.

There is growing public awareness of risk of methylmercury exposure associated with fish consumption (Hightower and Moore 2003; Knobeloch et al. 2005). Although omega-3 fatty acids in fish have been associated with healthful benefits, there is increasing evidence that the methylmercury content in some fish, if consumed too frequently, can lead to adverse health effects. The predominant concerns at this time include cardiovascular disease (Frustaci et al. 1999; Guallar et al. 2002; Rissanen et al. 2000; Salonen et al. 1995, 2000; Sørensen et al. 1999; Virtanen et al. 2005), autoimmune disease (Bagenstose et al. 1999; Bernier et al. 1995; Bigazzi 1994; Nielsen and Hultman 2002; Silva et al. 2004; Stejskal and Stejskal 1999; Stejskal et al. 1999; Via et al. 2003), infertility (Choy et al. 2002; Dickman and Leung 1998; Leung et al. 2001; Sheiner et al. 2003), neuropsychiatric effects (Beuter and Edwards 2004; Yokoo et al. 2003), and subjective complaints (Fukuda et al. 1999). Furthermore, many of these adverse health effects may occur at mercury levels previously thought to be safe.

The fetus has been the greatest concern because permanent damage to the developing brain can occur with methylmercury exposure. The U.S. Environment Protection Agency’s reference dose for mercury was based on a cord blood concentration of 5.8 μg/L and corresponds to a maternal intake of 0.1 μg Hg/kg body weight/day. However, it has been argued that this concentration should be lowered to 3.5 μg/L, based on more recent observations showing that cord blood mercury concentrations are approximately 70% higher than maternal concentrations (Stern and Smith 2003). It is of importance to note that mercury concentration across the placenta was not considered when the reference dose was established (Mahaffey 2005; Rice 2004; Rice et al. 2003).

Fish consumption accounts for most of the daily intake of mercury compounds in the United States, with lesser contributions from elemental mercury from mercury vapor in dental amalgams. Urine mercury is a reflection of inorganic or elemental exposure, as occurs with dental amalgams, magical uses of elemental mercury, and herbs and medicines adulterated with inorganic mercury. Only small amounts of methylmercury, which is consumed primarily through fish consumption, is metabolized to inorganic mercury and excreted in the urine (Dye et al. 2005).

There is also growing recognition within the medical community of the clinical importance of methylmercury exposure and of the connection between blood mercury levels and consumption of fish and shellfish products that are high in mercury. The American Medical Association (AMA) has advocated that physicians “assist in educating patients about the relative mercury content of fish and shellfish products and make patients aware of the advice contained in both national and regional consumer fish consumption advisories” (AMA 2004).

Since 1999, the National Health and Nutrition Examination Survey (NHANES) has reported total whole-blood mercury levels in children 1–5 years of age and in women 16–49 years of age. Recent reports based on NHANES data for 1999–2002 have examined blood mercury levels among the major racial/ethnic groups but have not examined blood mercury levels among those classified as “other” racial/ethnic group. The “other” group is of interest because it includes people at potentially high risk for methylmercury exposure through fish consumption, such as Asians, Pacific Islanders, and Native Americans (including Alaskan Natives) [Centers for Disease Control and Prevention (CDC) 2004, 2005; National Center for Health Statistics 2005; Sechena et al. 2003].

## Materials and Methods

This study complies with all applicable requirements of the U.S. human subject and research regulations. This project was reviewed by the Institutional Review Board of the California Pacific Medical Center and was declared exempt. Data were obtained from public access databases and contained no identifiers.

### Data sources.

NHANES is a complex, stratified, multistage probability cluster survey of a representative sample of the noninstitutionalized civilian population (CDC 2005; National Center for Health Statistics 2005).

We used data from the most recent NHANES (1999–2002) to compare blood mercury levels among women 16–49 years of age in the “other” racial/ethnic group (this includes all participants who self-identified as Asian, Pacific Islander, Native American, multiracial, or a race/ethnicity other than black, non-Hispanic white, Mexican American, or Hispanic) with those in all other racial/ethnic groups (CDC 2005; National Center for Health Statistics 2005).

### Study sample.

All women 16–49 years of age who were selected for both the interview and examination portions of NHANES and underwent whole-blood mercury testing were eligible for inclusion (*n* = 3,873). Among these, 236 participants had missing blood mercury results and were thus excluded from the analytic sample (*n* = 3,637). Among the women included in the analysis, 1,377 self-identified as non-Hispanic white, 1,106 as Mexican American, 794 as non-Hispanic black, and 220 as other Hispanic, and 140 were categorized as “other.”

### Statistical analysis.

We obtained population prevalence estimates for blood mercury levels ≥5.8 μg/L and ≥3.5 μg/L, respectively, using appropriate sample weights (Stata statistical software for complex survey design; StataCorp., College Station, TX) (CDC 2005; National Center for Health Statistics 2005). We compared the estimated prevalence of elevated blood mercury levels among participants in the “other” racial/ethnic group with those among Mexican Americans, non-Hispanic whites, and non-Hispanic blacks.

To determine whether observed differences in whole-blood mercury levels were most likely to reflect differences in dietary fish consumption rather than exposure to elemental or inorganic mercury, we examined self-reported fish consumption (number of fish and shellfish meals in the preceding 30 days) among female NHANES participants 16–49 years of age (*n* = 3,481) by racial/ethnic group. We also examined mean urine mercury-to-creatinine ratios (micrograms per gram) across racial/ethnic groups (*n* = 3,551). These analyses were also conducted using sample weights.

## Results

An estimated 16.59 ± 4.0% [mean ± SE; 95% confidence interval (CI), 8.41–24.77] of adult female participants who self-identified as Asian, Pacific Islander, Native American, or multiracial (*n* = 140) had blood mercury levels ≥5.8 μg/L, and an estimated 27.26 ± 4.22% (95% CI, 18.63–35.90) had levels ≥3.5 μg/L. In accordance with the NHANES analytical guidelines, these prevalence estimates are statistically reliable, because the relative SEs did not exceed 30% of the point estimates. The prevalence of elevated blood mercury levels in the “other” group is significantly higher than in all other racial/ethnic groups (Table 1).

Among the remaining participants (*n* = 3,497), an estimated 5.08 ± 0.90% (95% CI, 3.25–6.92) had blood levels ≥5.8 μg/L, and an estimated 10.86 ± 1.45% (95% CI, 7.90–13.83) had mercury levels ≥3.5 μg/L.

The mean number of fish and shellfish meals for the “other” racial/ethnic group was higher than for the remaining groups, although the 95% CIs overlapped with all except Mexican Americans. Fish and shellfish consumption among Mexican Americans is lower than among the “other” population. There were no statistically significant differences between groups in urine mercury:creatinine ratios (Table 2).

## Discussion

In the United States, 4.1 million people identified themselves as Alaskan Native or Native American alone or in combination with one or more races in Census 2000; 12.5 million people identified as Asian or Pacific Islander, with 51% residing in the West, 19% in the South, 12% in the Midwest, and 19% in the Northeast. Asians, Pacific Islanders, Alaskan Natives, and Native Americans accounted for approximately 6.0% of live births in 2001, or approximately 242,151 babies born (U.S. Census Bureau 2002a, 2002b; Ventura et al. 2003).

The prevalence of elevated blood mercury, whether this was defined as ≥5.8 μg/L or ≥3.5 μg/L, was significantly higher among members of the “other” racial/ethnic group than among any other racial/ethnic group and primarily reflected differences in organic mercury exposure, most likely due to fish consumption.

Although reports based on the most recent NHANES data from 2001–2002 have not reported mercury levels for this group (CDC 2004), NHANES data for the years 1999–2000 showed a non-statistically significant trend toward higher mercury levels in the “other” ethnic group (Mahaffey et al. 2004). Estimates presented here based on four years of NHANES data, 1999–2002, demonstrate that levels of mercury in this group are statistically significantly higher than for all other groups considered.

Our findings are consistent with those of smaller studies that showed a correlation of methylmercury through fish consumption within the racial/ethnic groups that comprise the “other” population of NHANES participants. The groups identified in these studies were Native peoples of the United States and Canada (Beuter and Edwards 2004; Bjerregaard and Hansen 2000; Clarkson 1976; Girard et al. 1996; Harnly et al. 1997; McKeown-Eyssen et al. 1983; Mergler et al. 1998; Muckle et al. 2001; Weihe et al. 2002) and residents of Hong Kong and China (Choy et al. 2002; Dickman and Leung 1998; Leung et al. 2001), Japan (Fukuda et al. 1999), and American Samoa (Marsh et al. 1974). In fact, a recent random-digit-dial fish consumption survey (with subsequent hair mercury levels in women 18–45 years of age from 12 states in the continental United States) found that Asians had methylmercury exposures greater than the reference dose 83% of the time, compared with 12% for the total survey population (Knobeloch et al. 2005).

Although NHANES was not designed to identify small subgroups at risk, it is imperative that significant populations at risk and important trends be identified. Such identification will help health care clinicians apply recommendations from the scientific literature to the patients they see in their communities, where appropriate.

## Conclusion

Study subjects in NHANES who self-identified as Asian, Pacific Islander, Native American, or multiracial had a higher prevalence of elevated blood mercury than all other racial/ethnic participants in the survey. It is important that both patients and clinicians be aware that members of this group are at increased risk for methylmercury exposure. Future studies should address reasons for the high mercury levels in this group and explore possible interventions for lowering risk of methylmercury exposure in this population.

## Correction

The standard error of 0.4% that appeared in the text and Table 1 of the original manuscript published online was incorrect. It has been corrected here to 4.0.

## Figures and Tables

**Table 1:** Prevalence of elevated blood mercury levels among women 16–49 years of age participating in NHANES 1999–2002 [mean ± SE (95% CI)].

Race/ethnicity	No.	≥3.5 μg/L (%)	≥5.8 μg/L (%)
Mexican American	1,106	5.71 ± 0.98 (3.70–7.72)	1.70 ± 0.41 (0.87–2.53)
Non-Hispanic white	1,377	11.29 ± 1.91 (7.40–15.19)	5.77 ± 1.24 (3.24–8.30)
Non-Hispanic black	794	12.47 ± 2.15 (8.08–16.90)	4.82 ± 1.49 (1.78–7.86)
Other Hispanic	220	10.38 ± 3.63 (2.95–17.81)	3.65 ± 1.87 (0.00–7.48)
Other^a^ race/ethnicity	140	27.26 ± 4.22 (18.63–35.90)	16.59 ± 4.0 (8.41–24.77)
All races/ethnicities except “other”	3,497	10.86 ± 1.45 (7.90–13.83)	5.08 ± 0.90 (3.25–6.92)
All participants	3,637	11.69 ± 1.42 (8.79–14.60)	5.66 ± 0.94 (3.75–7.58)

a Participants who self-identified as Asian, Pacific Islander, Native American, multiracial, or a race/ethnicity other than black, non-Hispanic white, Mexican American, or Hispanic.

**Table 2:** Fish/shellfish meals and urine Hg:creatinine ratios among women 16–49 years of age participating in NHANES 1999–2002 [mean ± SE (95% CI)].

Race/ethnicity	No. of fish/shellfish meals in the preceding 30 days	Urine Hg:creatinine ratio (μg/g)
Mexican American	2.86 ± 0.15 (2.55–3.17)	1.31 ± 0.12 (1.07–1.55)
Non-Hispanic white	4.63 ± 0.25 (4.13–5.14)	1.07 ± 0.06 (0.95–1.19)
Non-Hispanic black	4.90 ± 0.27 (4.35–5.45)	1.10 ± 0.12 (0.84–1.34)
Other Hispanic	3.82 ± 0.53 (2.74–4.90)	1.08 ± 0.11 (0.86–1.31)
Other^a^	8.02 ± 1.95 (4.03–12.02)	1.17 ± 0.14 (0.88–1.45)

a Participants who self-identified as Asian, Pacific Islander, Native American, multiracial, or as a race/ethnicity other than black, non-Hispanic white, Mexican American, or Hispanic.
